# Therapeutic Potential of *Lactobacillus rhamnosus* DS3316 via Cell Apoptosis in Colorectal Cancer

**DOI:** 10.4014/jmb.2505.05001

**Published:** 2025-07-18

**Authors:** Jinkwon Lee, Jeongmin Lee, In Hwan Tae, Yunsang Kang, Jinsan Kim, Sarang Kim, Haneol Yang, Kunhyang Park, Doo-Sang Park, Dae-Soo Kim, Hyun-Soo Cho

**Affiliations:** 1 Korea Research Institute of Bioscience and Biotechnology, Daejeon 34141, Republic of Korea; 2 Korea University of Science and Technology, Daejeon 34113, Republic of Korea; 3 Department of Biological Science, Sungkyunkwan University, Suwon 16419, Republic of Korea

**Keywords:** *Lactobacillus rhamnosus*, apoptosis, colorectal cancer

## Abstract

Colorectal cancer (CRC) has a very high mortality rate worldwide. Although various therapies have been developed to treat CRC, the need for novel therapeutic approaches has been increasing due to severe side effects and limited efficacy of current treatments. Recently, although research on the gut microbiome and its association with colon cancer has been growing, the mechanisms of gut microbiome inhibition in CRC remain insufficiently understood. Thus, in this study, we investigated the growth-inhibitory effects of the culture supernatant of *Lactobacillus rhamnosus* DS3316, isolated from infant feces, on CRC cell lines (HCT116 and SNUC5). And RNA-seq analysis revealed an increase in apoptosis-related terms induced by *L. rhamnosus* DS3316 treatment. Also, we found the non-toxicity of *L. rhamnosus* DS3316 in human iPSC-derived intenstine organoid. Thus, we suggested that *L. rhamnosus* DS3316 inhibits the growth of colorectal cancer cell lines without affecting normal cells. And *L. rhamnosus* DS3316 is expected to be a promising candidate for the development of microbiome-based colorectal cancer therapies. Furthermore, its combined use with various colorectal cancer treatment methods could lead to the proposal of more effective therapeutic approaches.

## Introduction

Colorectal cancer (CRC) is one of the leading causes of cancer-related deaths worldwide [1-3]. Surgical treatment is commonly employed for CRC, and chemotherapy agents such as capencitabine and 5-fluorouracil (5-FU) also used for increase of CRC treatment [4, 5]. Recently, targeted therapies (epidermal growth factor receptor (EGFR) inhibitors) [6] and immunotherapies (PD-1/PD-L1 inhibitors) [7] have shown enhanced treatment efficacy. However, due to significant side effects, low efficacy, and high recurrence rates, needs for the development of novel therapeutic approaches for CRC have been required.

Research on the relationship between gut microbiota and CRC has been growing in recent years. Several metabolites produced by gut microbiota, such as short-chain fatty acids (SCFAs), can inhibit the progression of CRC. Furthermore, various gut microbial species have been reported to play roles in suppressing CRC growth [8, 9], reducing drug resistance [10, 11], and enhancing the response to immunotherapy [12, 13]. Thus, these findings highlight the increasing importance of gut microbiota in CRC treatment. However, the safety aspects of microbiota and metabolites related to CRC, as well as studies on their mode of action (MOA), have not yet been fully understood.

*Lactobacillus rhamnosus*, a probiotic lactic acid bacterium, has recently garnered attention for its role in gut health and immune modulation [14]. As a Gram-positive facultative anaerobic bacterium, *L. rhamnosus* produces lactic acid through sugar fermentation, thereby maintaining the acidic environment of the gut [15]. Recent studies have also reported its relevance to various intestinal diseases [16]. Specifically, *L. rhamnosus* has been shown to inhibit the growth of CRC cell lines [17, 18]. Moreover, *Lactobacillus* cocktail suppressed the progression of CRC by modulating the bone morphogenetic protein (BMP) signaling pathway [19]. In addition, co-treatment with *L. rhamnosus* enhanced the anti-cancer effect of 5-fluorouracil (5-FU) in colorectal cancer cell lines [20]. Notably, *L. rhamnosus* had shown anti-cancer effects across multiple tumor types. In a primary liver cancer mouse model, the combination of *L. rhamnosus* and a dual PI3K/mTOR inhibitor led to the suppression of proinflammatory cytokine expression and inhibition of tumor growth, further highlighting the increasing relevance of *L. rhamnosus* as a potential therapeutic agent in diverse cancer treatments [21].

Thus, in this study, we identified the inhibitory effect of *L. rhamnosus* DS3316, isolated from infant feces, on CRC cell lines. Treatment with *L. rhamnosus* DS3316 supernatant (sup) increased apoptosis in CRC cell lines, and the induction of apoptosis was also observed in a 3D spheroid model that mimics an *in vivo* environment. Based on these findings, we report *L. rhamnosus* DS3316 as a novel probiotic strain with anti-colon cancer properties, demonstrating its ability to induce apoptosis in CRC cells. These results suggest the potential of *L. rhamnosus* DS3316 as a novel microbial therapeutic for CRC and indicate the possibility of synergistic effects when used in combination with other anticancer agents

## Materials and Methods

### Cell Culture

The colorectal cancer cell lines HCT116 and SNUC5 were obtained from the American Type Culture Collection (ATCC, USA) and the Korean Cell Line Bank (KCLB, Republic of Korea), respectively. The cells were maintained in RPMI-1640 medium (Cat. no. LM011-01, Welgene, Republic of Korea) supplemented with 10% fetal bovine serum (FBS; Cat. no. 10082147, Gibco, USA) and 1% penicillin/streptomycin (Cat. no. 15140122, Gibco). All experimental cultures were kept at 37°C under humidified conditions with 5% CO_2_.

### Bacterial Culture

The *L. rhamnosus* DS3316 strain was obtained from the Bio R&D Product program (https://biorp.kribb.re.kr/, BP1914267). The bacterial strain was cultivated in de Man, Rogosa and Sharpe (MRS) media (BD, USA) under anaerobic condition at 37°C for 36 h. The bacterial culture was incubated at 65°C for 30 min for Pasteurization and centrifuged at 3,000 g for 10 min. The supernatant was collected in a fresh new tube and kept at -70°C until use.

### 3D Spheroid Culture

To generate spheroid cultures, colorectal cancer cells were maintained in ultra-low attachment plates (Cat. no. 7007, Corning, USA). HCT116 and SNUC5 cells were plated at 5 × 10^4^ cells per well and allowed to grow for 24 h. Following initial incubation, cells were treated with *L. rhamnosus* DS3316 supernatant and maintained for 72 h. Spheroid formation was examined at 24-h intervals using an Olympus microscope (Cat. no. CKX53, Japan).

### Human Intestinal Organoid Culture

The human intestinal organoids (KCTC 3D 0011, passage 2) used in this study were obtained from the Korean Collection for Type Cultures (KCTC), supported by the Ministry of Food and Drug Safety under the project " Development of Organoid-Based Animal Alternative Resource Bank Establishment and Operation System" (RS-2024-00332162). The organoids were cultured in advanced DMEM/F12 medium (Cat. no. 12634010, Thermo Fisher Scientific, USA) supplemented with 100 ng/ml epidermal growth factor (Cat. no. 236-EG-200, EGF; R&D Systems), 500 ng/ml R-spondin1 (Cat. no. 4645-RS, R&D Systems, USA), 100 ng/ml Noggin (Cat. no. 6057-NG, R&D Systems), and 1X B27 supplement (Cat. no. 17504044, Thermo Fisher Scientific). The medium was refreshed every two days.

### Cell Viability Assay

Cells were seeded in 6-well plates at a density of 1 × 10^5^ cells per well (HCT116) or 2.5 × 10^5^ cells per well (SNUC5) and incubated overnight. After 72 h of *L. rhamnosus* DS3316 supernatant treatment, a mixture of Cell Counting Kit-8 (CCK-8; Cat. no. E-CK-A362, Elabscience, USA) solution and cell culture medium (1 ml/well) was added, followed by incubation at 37°C for 5 min. The absorbance was measured at 450 nm using a microplate reader. For crystal violet staining, cells were fixed with 100% methanol for 5 min and stained with 0.1% crystal violet solution (Cat. no. C0775, Sigma Aldrich, USA) [22].

### PI Staining

To evaluate cell death, spheroids were cultured for 24 h after seeding, thereafter being stained with propidium iodide (PI; Cat. no. P3566, Invitrogen, USA) and treated with *L. rhamnosus* DS3316 supernatant. PI-positive dead cells were observed using CELENA S Digital Cell Imaging System (Logos Biosystems, Republic of Korea). Spheroid morphology and cell death were analyzed through transmitted light and fluorescence imaging, respectively.

### Fluorescence-Activated Cell Sorting (FACS) Analysis

For analysis using the Muse Annexin V and Dead Cell Assay kit (Cat. no. MCH100105, Merck, Germany), the cells were collected and incubated in 20 min at room temperature. For analysis using the Muse Caspase 3/7kit (Cat. no. MCH100108, Merck), the cells were collected and incubated with caspase 3/7 reagent (Merck) for 30 min in a humidified atmosphere with 5% CO_2_ at 37°C. After incubation, the cells were incubated with Caspase 7-AAD (Merck) for 5 min at room temperature. After incubation, ~ 1 × 10^5^ cells were analyzed using a Muse Cell analyzer (Merck). The FACS results were analyzed using Muse 1.6 Analysis software (Merck).

### RNA Sequencing Analysis

For total RNA-seq analysis, using TrueSeq RNA Sample Preparation Kit V2, purification and library construction were carried out with total RNA, and Illumina NextSeq 1000 machines (Illumina, 20038898) were used for sequencing, with a read length of 2 × 100 bases. A filtered read set was created using the Cutadapt v1.18 (https://cutadapt.readthedocs.io/en/stable/) command line parameters ‘-a AGATCGGAAGAGCACACGTCT GAACTCCAGTCAC -AAGATCGGAA GAGCGTCGTGTAGGGAAAGAGTGTA -m 50 -O 5’, and Sickle v1.33 (https://github.com/najoshi/sickle) was used to remove the low-quality sequence (Phred score < 20) to a minimum length of 50 bp. We assessed the quality of the paired-end reads using FastQC version 0.11.4. Additionally, duplicate sequences were examined through the application of the FASTQC tool. The trimmed data containing low-quality reads and poly-N sequences were processed using the NGSQCToolkit v2.3.3 (https://github.com/mjain-lab/NGSQCToolkit). The reads were subsequently aligned to the human genome assembly GRCh38.97 (Accession No. GCA_000001405.27) by HISAT2 v2.1.0 (https://daehwankimlab.github.io/hisat2/). The obtained transcripts were quantified in fragments per kilobase million (FPKM) format using StringTie v2.2.1 (https://github.com/gpertea/stringtie) to calculate expression values and obtain normalized counts.

### Statistical Analysis

The results are expressed as the means ± SDs (error bars). Comparisons between two groups were conducted using an unpaired t test. A *p*-value < 0.05 was statistically signifcant.

## Result

### Confirmation of Cell Growth Inhibition by *L. rhamnosus* DS3316 Supernatant (sup) Treatment

**Fig. 1:**
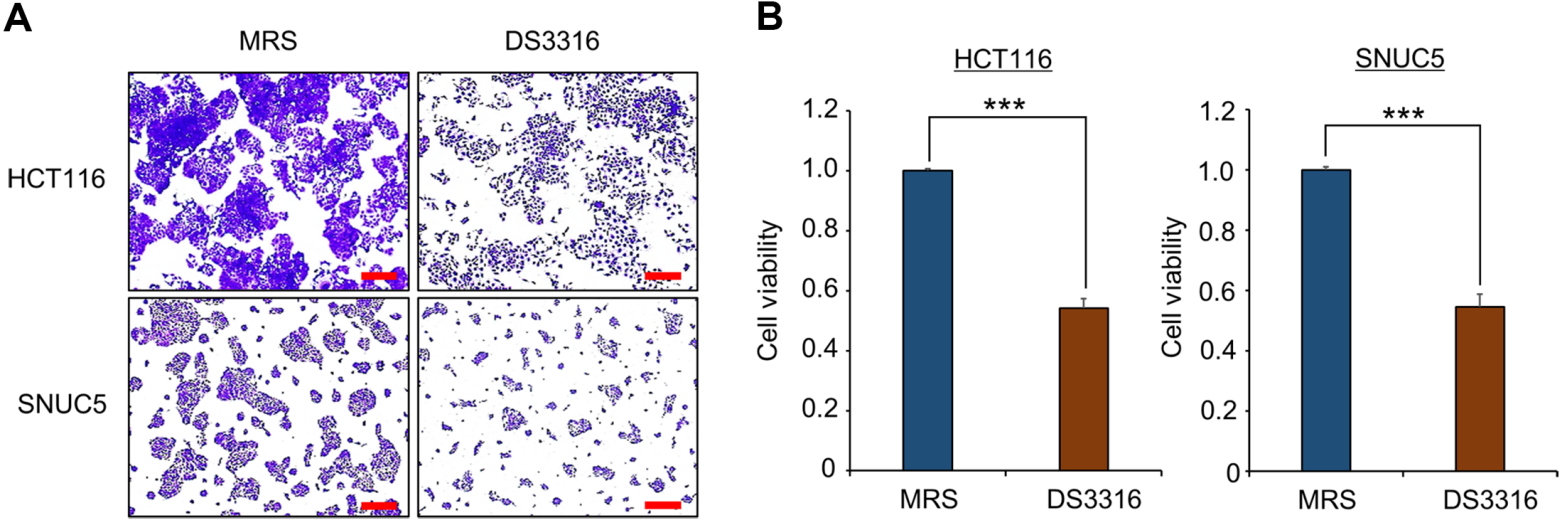
*L. rhamnosus* DS3316 treatment inhibited cell growth in HCT116 and SNUC5 cell lines. (**A**) Cell growth assay after *L. rhamnosus* DS3316 treatment for 72 h. HCT116 and SNUC5 cells were fixed in 100% methanol and stained with crystal violet solution. Scale bars 200 μm. (**B**) CCK-8 assay after *L. rhamnosus* DS3316 treatment for 72 h. HCT116 and SNUC5 cells were incubated for 5 min at 37°C after addition of CCK-8 solution. Cell viability was measured using a microplate reader (450 nm). The mean ± of three independent experiments is shown. P values were calculated using Student’s *t*-test (***p < 0.001).

**Fig. 2:**
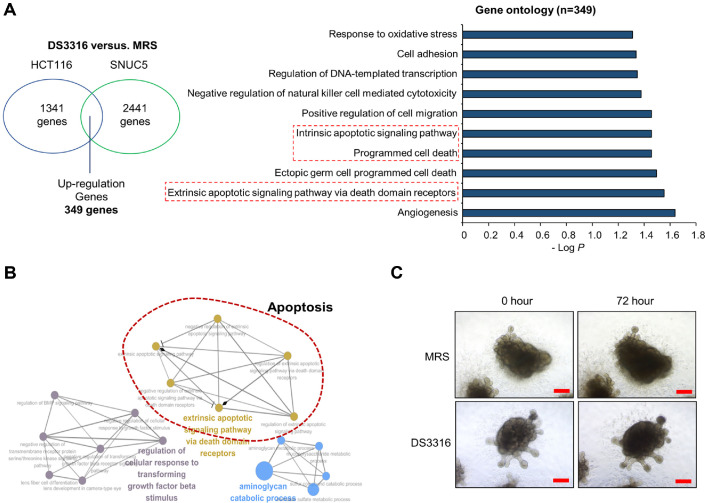
GO term analysis demonstrate apoptosis induction by *L. rhamnosus* DS3316 treatment in HCT116 and SNUC5 cell lines. (**A**) and (**B**), DAVID (**A**) and ClueGO (**B**)-based GO analysis of the RNA-seq results, among the 349 upregulated genes. Enriched GO terms are shown. (**C**) Human intestinal organoids after *L. rhamnosus* DS3316 treatment for 72 h. Scale bars 200 μm.

To evaluate the growth-inhibitory effect of *L. rhamnosus* DS3316 sup on CRC cell lines, we treated HCT116 and SNUC5 cells with *L. rhamnosus* DS3316 sup. The crystal violet (CV) staining demonstrated that *L. rhamnosus* DS3316 sup treatment suppressed the growth of CRC cells (Fig. 1A). Similarly, the Cell Counting Kit-8 (CCK-8) assay confirmed the inhibitory effect of *L. rhamnosus* DS3316 sup on CRC cell growth, consistent with the CV staining results (Fig. 1B). Next, to investigate the mechanism underlying the growth inhibition by *L. rhamnosus* DS3316, RNA-seq analysis was performed on HCT116 and SNUC5 cell lines after treatment with *L. rhamnosus* sup. Gene Ontology (GO) term analysis revealed that a strong association between *L. rhamnosus* DS3316 and apoptosis-related terms, such as “intrinsic apoptotic signaling pathway, programmed cell death, extrinsic apoptotic signaling pathway via death domain receptors” and “Apoptosis” observed after treatment of *L. rhamnosus* DS3316 (Fig. 2A and 2B). Furthermore, to determine whether *L. rhamnosus* DS3316 sup affects normal cell growth, we evaluated its effect on human pluripotent stem cell-derived intestinal organoids (hIOs). As shown in Fig. 2C, no changes in hIO growth were observed following *L. rhamnosus* DS3316 sup treatment. Thus, we suggested that *L. rhamnosus* DS3316 sup selectively induces apoptosis in CRC cells, leading to growth inhibition, without affecting normal cells.

### *L. rhamnosus* DS3316 Sup Induces Cell Apoptosis in CRC Cell Lines

**Fig. 3:**
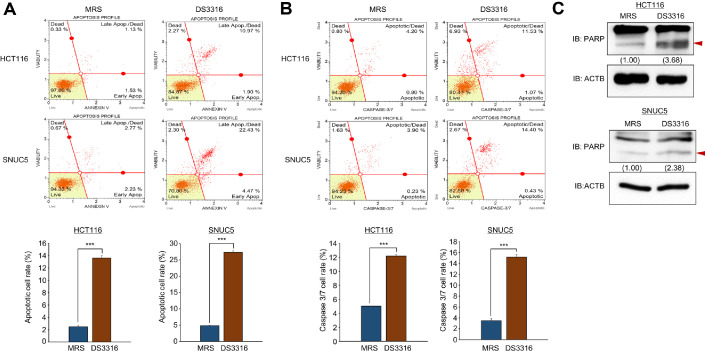
*L. rhamnosus* DS3316 treatment induced cell apoptosis. (**A**) FACS analysis of Annexin V staining was conducted after *L. rhamnosus* DS3316 treatment for 72 h. The lower right and upper right quadrants indicate early apoptosis and late apoptosis (upper), respectively. The quantification of apoptosis. Mean ± SD of three independent experiments. The P values were calculated using Student’s t‐test (***P < 0.001). (**B**) FACS analysis using Muse Caspase 3/7 working solution was conducted after *L. rhamnosus* DS3316 treatment for 72 h. The upper right panel indicates the apoptotic and dead cell proportions (upper). The quantification of caspase‐3/7 activity. Mean ± SD of three independent experiments. The P values were calculated using Student’s t‐test (***P < 0.001). (**C**) Western blot analysis after *L. rhamnosus* DS3316 treatment using anti- PARP antibody. ACTB was used as the internal control in HCT116 and SNUC5 cells. The signal intensities were quantified using ImageJ software.

To confirm the apoptosis related terms by *L. rhamnosus* DS3316 sup on CRC cell lines in GO analysis, FACS analysis was conducted. Treatment of HCT116 and SNUC5 cells with *L. rhamnosus* DS3316 sup resulted in a significant increase in the late apoptosis population compared to the negative control (MRS treatment) (Fig. 3A). Additionally, caspase 3/7 activity showed a marked increase in caspase 3/7 activity following *L. rhamnosus* DS3316 sup treatment compared to MRS treatment (Fig. 3B). This result indicated that *L. rhamnosus* DS3316 sup promotes cell apoptosis in CRC cell lines. Moreover, western blot analysis confirmed apoptosis induction, as shown by the increased status of cleaved PARP after *L. rhamnosus* DS3316 sup treatment (Fig. 3C). Thus, we could suggest that *L. rhamnosus* DS3316 sup inhibits cell growth by inducing apoptosis in CRC cell lines.

### Growth Inhibition by *L. rhamnosus* DS3316 Sup in 3D Model

**Fig. 4:**
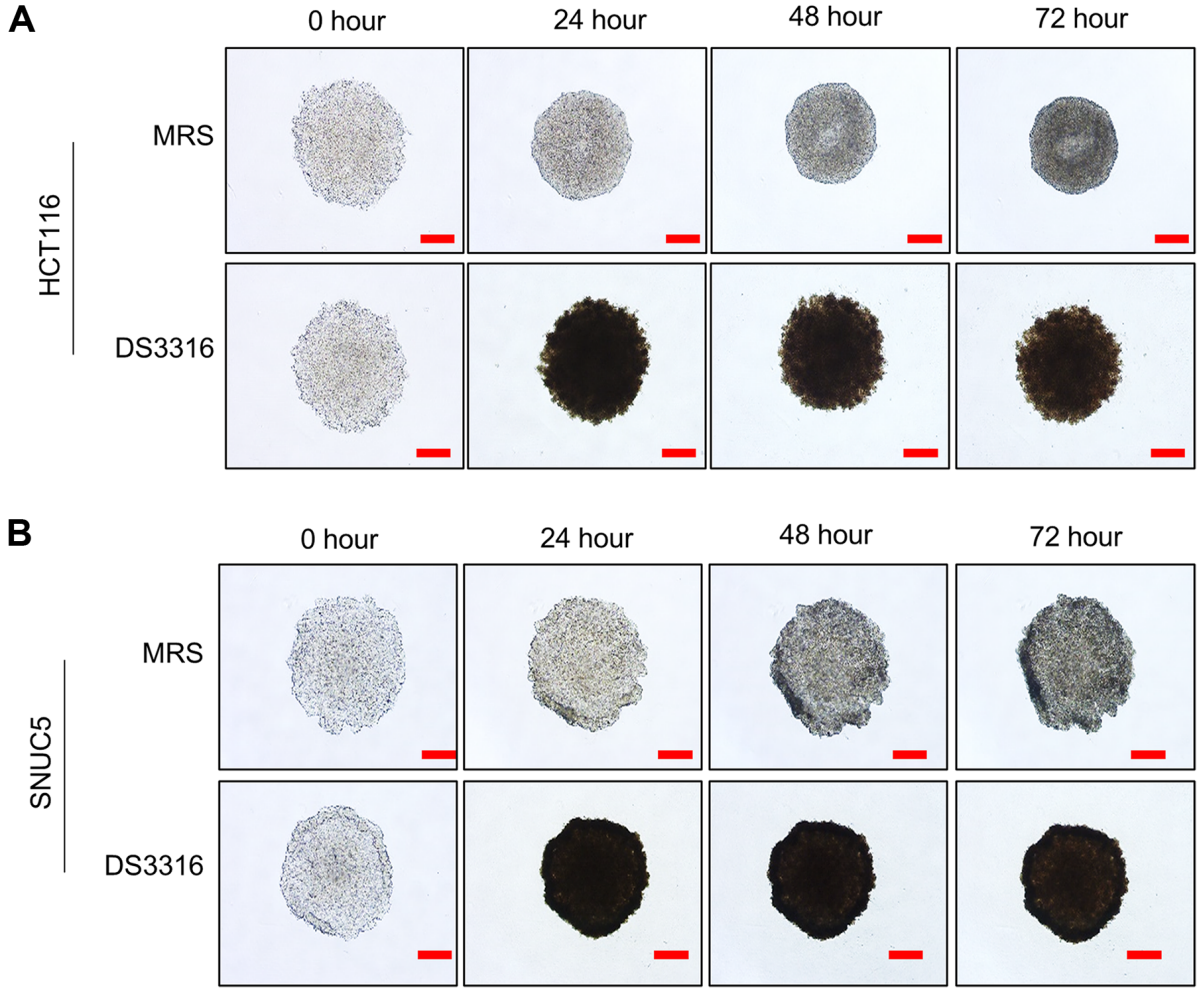
*L. rhamnosus* DS3316 treatment reduced aggregation of 3D spheroid culture system. (**A**) 3D spheroid formation assay. *L. rhamnosus* DS3316 treatment was performed in HCT116 cells for 72 h. Cells were loaded onto ULA plates. (**B**) 3D spheroid formation assay. *L. rhamnosus* DS3316 treatment was performed in SNUC5 cells for 72 h. Cells were loaded onto ULA plates. Scale bars 200 μm.

**Fig. 5:**
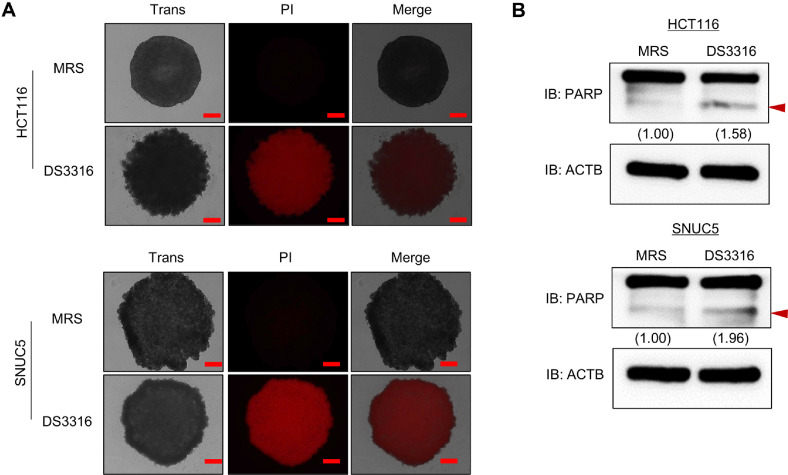
*L. rhamnosus* DS3316 treatment induced cell apoptosis in 3D spheroid culture system. (**A**) After 72 h of *L. rhamnosus* DS3316 treatment, PI staining was conducted. Scale bars 200 μm. (**B**) Western blot analysis after *L. rhamnosus* DS3316 treatment using anti-PARP antibody. ACTB was used as the internal control in HCT116 and SNUC5 cells. The signal intensities were quantified using ImageJ software.

Three dimensional (3D) cultures of cancer cells are reported to better mimic the structural and physiological characteristics of tumors *in vivo* (ref). To establish a more *in vivo*-like environment, HCT116 and SNUC5 cells were cultured using ultra-low attachment (ULA) plates, successfully forming 3D spheroids (Fig. 4A and 4B). Treatment of 3D spheroids with *L. rhamnosus* DS3316 sup revealed an inhibitory effect on cell growth, as indicated by reduced spheroid aggregation (Fig. 4A and 4B). Additionally, apoptosis in 3D spheroids was assessed using PI (Propidium Iodide) staining. Following *L. rhamnosus* DS3316 sup treatment, late apoptosis signals were significantly increased (Fig. 5A). Similar to the results observed in 2D cultures, *L. rhamnosus* DS3316 sup treatment also induced apoptosis in the 3D spheroid model. Next, western blot analysis further confirmed increased cleaved PARP status in *L. rhamnosus* DS3316 sup-treated spheroids compared to the MRS-treated control (Fig. 5B). These results suggested that *L. rhamnosus* DS3316 sup effectively induces cell apoptosis and inhibits cell growth even in an *in vivo*-mimicking 3D spheroid model. Thus, we could demonstrate that *L. rhamnosus* DS3316 sup selectively induces apoptosis in CRC cells, leading to growth inhibition both in 2D and 3D culture models. These results highlight the potential of *L. rhamnosus* DS3316 as a novel microbial therapeutic for CRC treatment, with the possibility of synergistic effects when combined with existing anticancer agents.

## Discussion

Various microbial metabolites, such as short chain fatty acids (SCFAs) play a critical role in inhibiting the growth of CRC. As a result, screening microbiota involved in suppressing CRC has become a crucial step in developing effective microbiome therapies in CRC treatment. Thus, here, we demonstrated the anti-growth effects of *L. rhamnosus* DS3316, isolated from infant feces. Treatment with the culture supernatant of *L. rhamnosus* DS3316 inhibited the cell growth of two colon cancer cell lines (HCT116, SNUC5), suggesting its potential as an important therapeutic microbiota for the treatment and prevention of CRC. This research highlights the ability of *L. rhamnosus* DS3316 to suppress the growth of CRC cell lines, while also confirming its safety using normal human intestinal organoids (hIOs). The treatment with *L. rhamnosus* DS3316 did not affect the growth of normal intestinal cells but significantly inhibited the growth of 2D- and 3D-cultured CRC cell lines. Thus, we suggested that *L. rhamnosus* DS3316 selectively targets CRC cells without impacting normal cells. However, to enable clinical application of *L. rhamnosus* DS3316, further analysis is required to identify the specific metabolites in its culture supernatant responsible for inhibiting CRC growth.

Recent studies have reported that SCFAs produced by gut microbiota are effective in suppressing CRC progression [23]. Thus, future studies should focus on the metabolite profiling of *L. rhamnosus* DS3316 to identify the active components. Additionally, research on the mode of action (MOA) of single metabolites in inducing apoptosis in CRC cells is essential. In addition, *in vivo* studies using the colorectal cancer-suppressive metabolites identified through metabolite profiling will be necessary to further validate the anti-cancer effects of *L. rhamnosus* DS3316. Furthermore, it may be possible to evaluate its impact on chemoresistance, which is commonly observed in colorectal cancer. Through these approaches, *L. rhamnosus* DS3316 could be demonstrated to not only suppress tumor growth but also overcome chemoresistance, thereby providing important foundational data for the development of microbiome-based therapeutics for colorectal cancer. And for more effective CRC treatments, a microbial cocktail comprising various CRC-suppressing microbiota could be developed.

In this study, we evaluated the safety of *L. rhamnosus* DS3316 using hIOs. While normal epithelial cell lines could be used to assess safety of *L. rhamnosus* DS3316, hIOs could use a more accurate model, as they contain a diverse array of intestinal cell types. Although the small intestinal organoids used in this study differ from colonic tissue in cell-type distribution, they still include similar cell types, such as goblet cells. Therefore, hIOs provided a more physiologically relevant platform than a single normal epithelial cell lines for evaluating safety. Through this approach, we confirmed the safety of *L. rhamnosus* DS3316, supporting its potential as a therapeutic candidate for colorectal cancer. Thus using hIOs, the toxicity of such microbial cocktails could be tested at different doses, paving the way for safer microbial therapies for CRC.

In conclusion, this study confirmed that the culture supernatant of *L. rhamnosus* DS3316 induced apoptosis and inhibited the growth of two CRC cell lines, HCT116 and SNUC5. These findings suggested the potential application of *L. rhamnosus* DS3316 in the development of microbial therapeutics for CRC. Furthermore, combination therapy with existing anti-cancer drugs could yield synergistic effects, enabling the development of microbial therapeutics that are both more effective and less prone to side effects.
